# Development and Feasibility of a Prehabilitation Protocol for Patients Diagnosed with Head and Neck Cancer

**DOI:** 10.7759/cureus.9898

**Published:** 2020-08-20

**Authors:** Lori Boright, Deb J Doherty, Christopher M Wilson, Sara K Arena, Carlos Ramirez

**Affiliations:** 1 Physical Therapy, Oakland Univeristy, Rochester, USA; 2 Physical Therapy, Oakland University, Rochester, USA; 3 Rehabilitation Services, Beaumont Health, Troy, USA; 4 Head and Neck Surgery, Ascension St. John Hospital, Detroit, USA

**Keywords:** physical therapy, cancer rehabilitation, oncology rehabilitation, exercise, neck dissection, range of motion, aerobic capacity

## Abstract

Background: Head and neck (H&N) cancers account for 4% of total cancers diagnosed. However, quality of life (QoL) implications are more severe for this patient population due to the complexity, extent, and deformities resulting from treatment interventions. Principally debilitating complications include diminished functional walking capacity, reduced cervical range of motion (ROM), and scapular strength. An extensive literature search revealed a paucity of evidence utilizing physical therapy assessment and intervention for this population. The purpose of this study was to describe the development and clinical feasibility of a prehabilitation program aimed to thwart these complications for patients diagnosed with H&N cancer.

Methods: Inclusion criteria: male or female, 18+ years, speak and read the English language, ambulate independently, diagnosed with H&N cancer, and scheduled for surgical intervention. Institutional Review Board approval was obtained. Pre- and post-surgical measurements included the six-minute walk test (6MWT), cervical ROM, manual muscle testing for scapular strength, and three questionnaires: physical activity history, health behaviors questionnaire, and the Functional Assessment Cancer Therapy H&N QoL survey.

Results: Three participants were enrolled (two males and one female) all identifying as Caucasian and between 60-90 years of age. Pre- to post-cervical ROM demonstrated decline in extension/bilateral rotation for two of three participants. Two participants demonstrated decreased 6MWT distance while one increased. No participants experienced any adverse effects of the prehabilitation program.

Conclusion: This is the first study protocol to describe a physical therapist-administered H&N cancer prehabilitation program. Professionally administered education and exercise has potential to prevent, manage, and mitigate the adverse effects of cancer treatment. Additional research is needed to define the importance of prehabilitation relative to improved clinical outcomes and improved QoL. Patients with a cancer diagnosis are susceptible to impairments and functional limitations as a result of treatments and this prehabilitation program demonstrates potential to positively impact outcomes across the survivorship continuum. Due to their education and integration within the medical system, physical therapists are well-positioned to lead the effort to unify theory and clearly define parameters for oncology prehabilitation.

## Introduction

The National Cancer Institute predicts 53,260 new diagnoses of head and neck (H&N) cancers in 2020 in the United States [1]. Head and neck cancers, those of the paranasal sinuses, nasal and oral cavities, salivary glands, pharynx, larynx, and upper cervical lymph nodes, account for 4% of total cancers diagnosed [2]. Though H&N cancers account for a small percentage of cancers diagnosed, quality of life (QoL) implications are more substantial for this patient population [3]. Patients often are left with varying degrees of facial and upper body anatomical/structural changes resulting not only from the cancer but also from invasive treatment interventions. As a result of the aforementioned changes, patients may experience a deterioration in perceived QoL, which impacts the patient’s physical, social, emotional, and functional well-being [3].

“Prehabilitation is a process on the cancer continuum of care that occurs between the time of cancer diagnosis and the beginning of acute treatment and includes physical and psychological assessments that establish a baseline functional level, identify impairments, and provide interventions that promote physical and psychological health to reduce the incidence and/or severity of future impairments [4].” Providing education, exercise/physical activity, and self-treatment strategies to patients as part of a prehabilitation program has the potential to assist not only in ensuring program compliance but also in maximizing outcomes. Patients who receive these interventions often experience improved outcomes as compared to those who do not participate in prehabilitation programming [5].

Strong evidence demonstrates the physiological benefits of exercise/physical activity to treat the adverse effects caused by cancer and its treatments; these benefits include reducing cardiopulmonary impairment, optimizing pain control, and reducing the risk of acquiring comorbidities [6]. Recent research has shown that exercise/physical activity is a cost-effective, easy to administer, non-pharmacological treatment [4]. Exercise/physical activity is efficacious in managing the adverse effects of cancer and its treatments and is a key intervention for many cancers including H&N cancers [4,7].

The multitude and complexity of the adverse effects of cancer and its treatments compromise body systems (neurological, musculoskeletal, cardiopulmonary), each of which can be treated by physical therapists (PTs). Physical therapists are well-positioned to identify and to address these adverse effects because they understand their interrelated nature on body structures and function. Prehabilitation aims to prevent and decrease the intensity of rather than to correct or manage adverse effects. To establish the role of H&N cancer prehabilitation, the providing clinician must have a comprehensive appreciation for and understanding of the disease process, its treatments, and associated adverse effects.

Surgery is typically the primary intervention for the treatment of patients diagnosed with H&N cancers [7]. The trauma from surgery often negatively impacts proximal tissues which have the potential to negatively impact a number of global physiological systems as a result of immobility [8]. Swelling of the face and neck and potential nerve damage may cause further motor, sensory, and functional deficits, such as diminished neck and/or shoulder range of motion (ROM). Significant QoL issues result from this sequelae, causing difficulty managing both basic and advanced activities of daily living (ADLs) [9]. In partnership with speech language pathologists (SLP) who address potential swallowing and speech limitations, PTs are often consulted to address the negative sequelae of H&N cancer and its treatments.

Radiation therapy (RT) often is a second line of intervention and is typically introduced as a treatment option post-surgically. Adverse skin effects commonly reported and diagnosed during and after exposure to radiation include: diminished integrity, redness, irritation, skin sensitivity, or texture changes [10]. Radiation-induced fibrosis, skin induration/thickening, muscle shortening, atrophy, limited joint mobility, lymphedema, mucosal fibrosis, ulceration, fistula, hollow organ stenosis, nerve damage, and pain also may result [10]. Decreased appetite, which can negatively impact the nutritional status of a patient, is also a common occurrence. Other potential adverse effects of RT include dry mouth, otalgia, trismus (diminished mouth opening), and fatigue, all of which can negatively affect functional status and participation. These adverse effects can occur during the actual treatment course itself or may develop months or years after treatment has ceased [11]. Cancer-related fatigue, the most reported adverse effect from cancer treatment, is an example. Physical therapists are positioned with their specialty skill set to educate and to treat these adverse effects as well.

Chemotherapy is presently viewed as an adjunctive treatment for patients diagnosed with H&N cancer, though it is less common and typically reserved for locally advanced recurrent and metastatic diagnoses [7]. Chemotherapy is usually prescribed post-surgically and may be administered concurrently with or after RT [7]. Common adverse effects are wide-ranging due to the systemic nature of chemotherapy and include fatigue, nausea, malaise, cardiotoxicity, cognitive impairment, neuropathy, and alopecia [7]. Once again, PTs also are well-positioned and trained to effectively address preventative measures and management of the above adverse effects, thereby improving the QoL for all cancer survivors. 

The multitude of potential and aforementioned adverse effects result in common rehabilitation-related impairments that include but are not limited to restricted cervical ROM, diminished scapular strength and impaired posture, as well as reduced functional walking capacity and increased risk of cardiovascular disease [11]. The long-term impact of reduced physical functioning, impaired swallowing function, speech production, and reduced sensation relative to taste and smell all have a negative impact on long-term QoL [12]. Oral and jaw function impairments result in insecurities related to social eating and other social contact that further negatively impact QoL and promote social isolation [12]. 

There is growing evidence of the efficacy for prehabilitation in persons diagnosed with colorectal [13], prostate [14], gastrointestinal [15], and lung [16] cancers that include unimodal and multimodal interventions. There were no articles identified that examined prehabilitation in patients diagnosed with H&N cancers provided by PTs. Multimodal studies, those including exercise, nutrition, and psychosocial interventions, demonstrated a higher success rate toward achieving positively correlated statistical significance for their interventions and assessments [15,16]. The preferred mode of cardiovascular exercise utilized was walking and incorporating strengthening exercises and breathing exercises, resulted in more positive clinical outcomes [13]. Relative to exercise delivery, supervised exercise sessions were found to enhance compliance, whereas unsupervised sessions typically were included out of a necessity to achieve frequency and encompassed the home exercise program (HEP). The frequency of exercise varied across all studies from two to five times per week, and the duration of the prehabilitation period varied from one to four weeks as a result of limited time frames between diagnosis and onset of medical treatment interventions. Inclusion of a HEP resulted in improved outcomes overall and afforded some control to patients during a time in their lives when they typically experience little control given the complex surgical and adjunctive medical interventions frequently required. The preferred measure of functional walking capacity present in the literature was the six-minute walk test (6MWT) and overall was found to improve in the majority of prehabilitation intervention groups across all studies [15]. Quality of life was found to improve in the groups receiving the pre-treatment exercise intervention [15]. Incorporating nutritional and psychosocial interventions in a prehabilitation program generally enhanced positive outcomes [15]. 

Prehabilitation interventions have successfully yielded beneficial outcome measures for many other cancer diagnoses including colorectal, lung, bladder, breast, prostate, endometrial, and liver diagnoses. These outcomes include functional walking capacity, maximal oxygen consumption (VO_2_MAX), and QoL.

Only one H&N cancer prehabilitation publication was found and it was entitled: “Prophylactic swallowing exercises in patients with H&N cancer undergoing chemoradiation [17].” This randomized control trial (RCT) had a sample size of 26, with 13 patients included in the intervention group and 13 in the control group. The prehabilitation intervention included supervised swallowing exercise training and a HEP taught by an SLP prior to and throughout chemoradiation therapy for the intervention group. The exercises included the effortful swallow, the super supraglottic swallow, the tongue hold maneuver, tongue retraction, and the Mendelsohn Maneuver. The primary outcomes examined were swallowing function and swallowing QoL, which demonstrated favorable results. Specifically, the intervention group showed statistically significant improved swallowing function at the three- and six-month follow-up assessments. Researchers noted that 69% (nine of 13) of patients in the intervention group effectively were unable to complete the entire exercise protocol throughout protocol duration and is a limitation of the study. Oral pain, throat discomfort, and overall fatigue brought about by chemoradiation therapy were cited as primary reasons that patients could not complete the study protocol.

This study neglected to address many of the associated functional complications that, in other populations, were successfully mitigated by interventions related to ROM, strength, and endurance. However, a paucity of evidence is published in the professional literature related to prehabilitation for persons diagnosed with H&N cancers. The literature presently lacks clear recommendations related to exercise frequency, duration, and mode among all cancer prehabilitation studies. Finally, evidence of associated QoL impact, psychosocial issues, and cognitive impairment resulting from cancer treatments is sparse in the available literature. The primary purpose of this study was to describe the development of a multimodal prehabilitation study protocol which includes physical therapy intervention in patients diagnosed with H&N cancer. The secondary purpose was to report feasibility study results from patients diagnosed with H&N cancer.

## Materials and methods

Protocol development

As the prior literature review did not reveal PT guided protocols for the H&N cancer population, protocols utilized with other cancer diagnoses informed the development of the protocol used in this inquiry. Its multimodal design was selected because of the high value of evidence provided and increased prevalence of positively correlated results with more than one intervention in the existing literature. Following positive outcomes from previous prehabilitation studies with various cancer diagnoses, inclusion of interventions for ROM, strength, endurance, and QoL was warranted in addition to nutritional interventions. Another objective of this project was to concept test the protocol developed. A convenience sample of three individuals diagnosed with H&N cancer and scheduled for surgical intervention with one H&N cancer surgeon was used. 

Inclusion criteria included: male or female patient, aged 18+ years, recently diagnosed with H&N cancer, and scheduled to undergo surgical intervention. These surgeries included selective neck dissection, modified radical neck dissection, or radical neck dissection. Participants also were required to speak and read the English language and ambulate independently, with or without the use of an assistive device. Participants were excluded in the event of metastatic disease to the brain or bones or if deemed unsafe for participation by their attending physicians.

Recruitment

Screening for eligibility was performed by the H&N nurse navigator and/or surgical resident team. A study flyer was present in the physician’s office to promote recruitment efforts. Upon verification of inclusion and exclusion criteria, written consent was obtained and the investigation visit was completed. To protect the rights of participants, informed consent was secured prior to study enrollment.

Protocol Timeline

**Figure 1:**
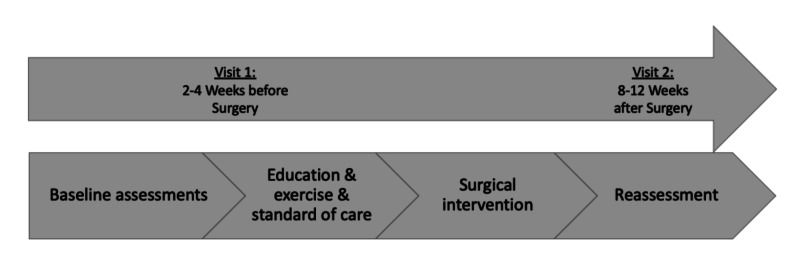
Protocol Diagram

The timeline for data collection is represented in Figure 1 and includes visit one (the prehabilitation visit), which was completed two to four weeks prior to surgical intervention, and visit two (the post-surgical visit), which was completed eight to 12 weeks following the surgical procedure. 

Data Collector Preparation and Skill Validation

**Figure 2:**
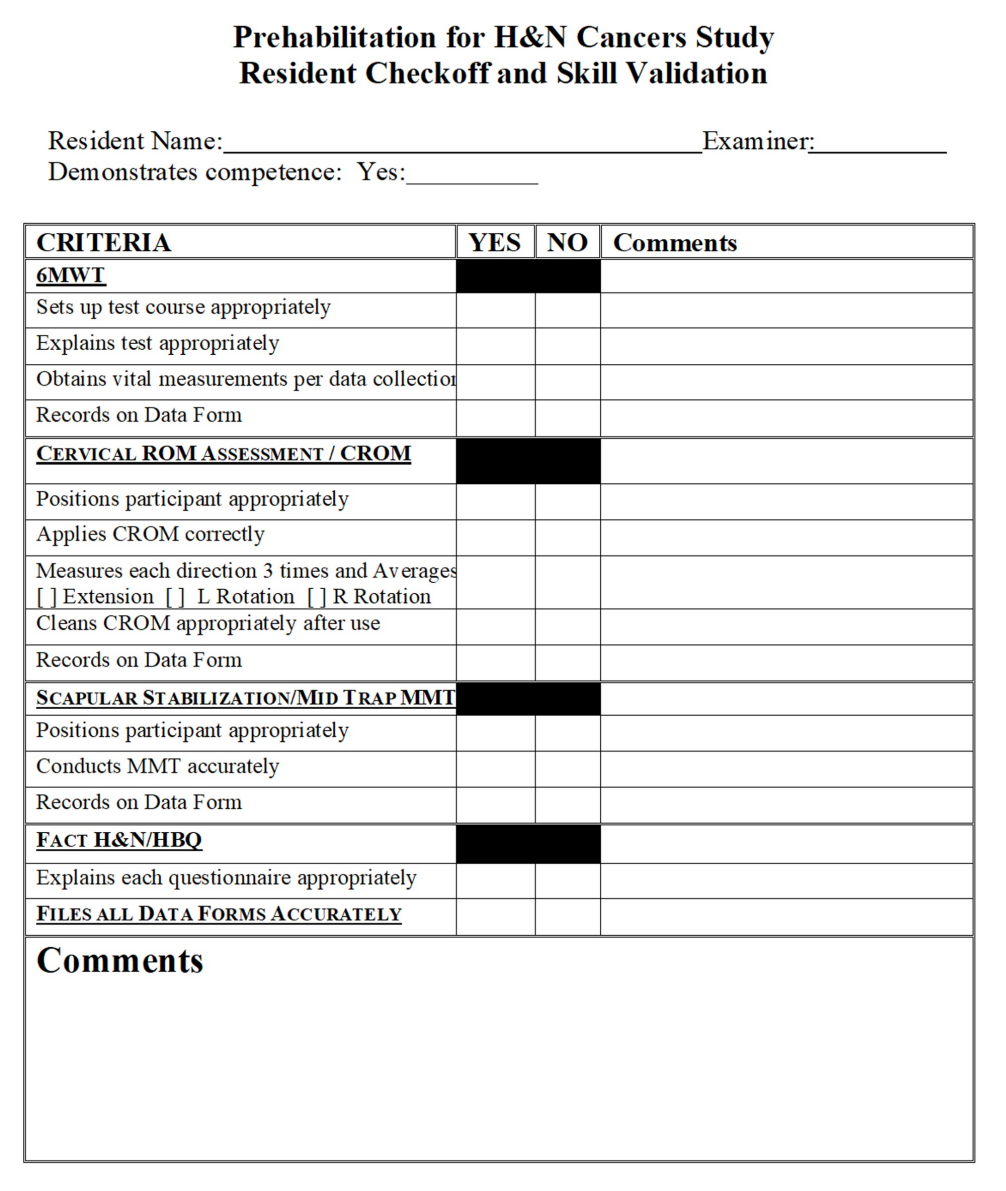
Resident Training Competency Rubric CROM=cervical range of motion, H&N=head and neck, HBQ=Health Behaviors Questionnaire, 6MWT=six minute walk test, MMT=manual muscle testing

Prior to data collection, the principal investigator conducted a four-hour training session on the use, function, and logistical administration of all six data collection measurement tools. This training was provided to one of the surgeon’s oncology residents and the H&N nurse navigator (a registered nurse, who works exclusively with the surgeon). A competency rubric (Figure 2) was created with the assistance of a content expert. Skill validation was conducted at the conclusion of the four-hour training session to ensure competency. 

Data Collection 

Data collection consisted of six assessments. The first of these was the Health Behaviors Questionnaire (HBQ) [18]. The HBQ assists in quantifying current behaviors and determining willingness to change in four domains. The HBQ includes self-reported engagement in performing regular physical activity, consuming recommended amounts of fruits and vegetables, smoking habits, and maintaining a healthy weight. The questionnaire defines healthy weight as having a body mass index between 18 and 25, which is consistent with the Center for Disease Control and Prevention recommended criteria [19]. 

**Figure 3:**
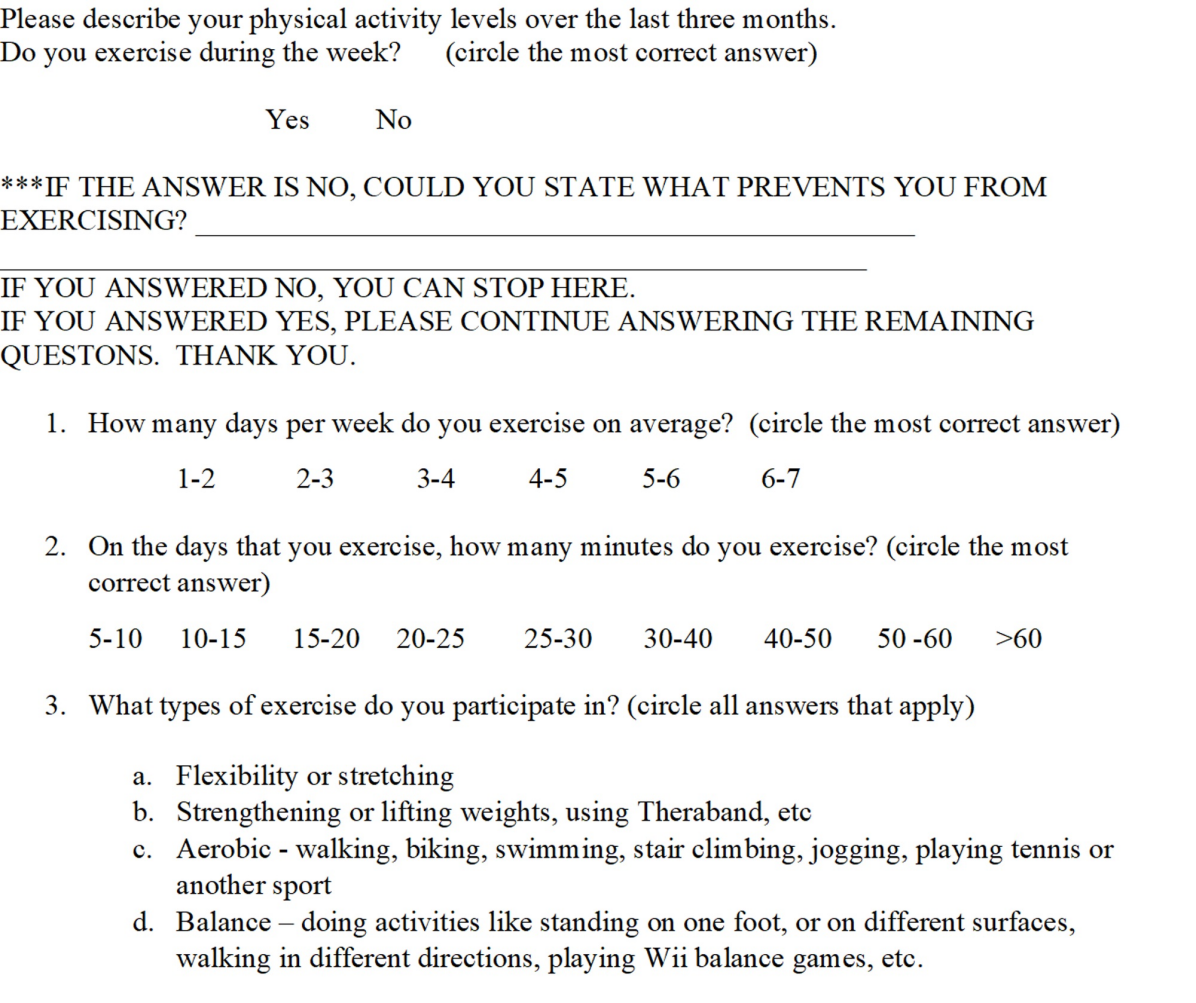
Physical Activity History (PA HX)

The Physical Activity History (PA History) (Figure 3) also was included in the assessment and was developed by the research team to describe physical activity habits over the previous three months. This tool has not been validated. A respondent answering “yes” to the question of “Do you exercise during the week?” was prompted to answer questions of “How many times per week?” “How many minutes per session?” and “What type of exercise do you participate in?” Respondents indicating “no” to the question of “Do you exercise during the week?” were prompted to indicate what was preventing them from exercising.

The Functional Assessment of Cancer Therapy - Head & Neck (FACT H&N) survey was also conducted and is a valid and reliable self-administered QoL survey utilizing a 0-4 point ordinal scale. A total of 39 questions quantify QoL relative to five domains: Physical Well-Being, Social/Family Well-Being, Emotional Well-Being, Functional Well-Being, and Additional Concerns that are specific to H&N cancer survivors. Internal consistency was reported as .63-.89 [20].

**Figure 4:**
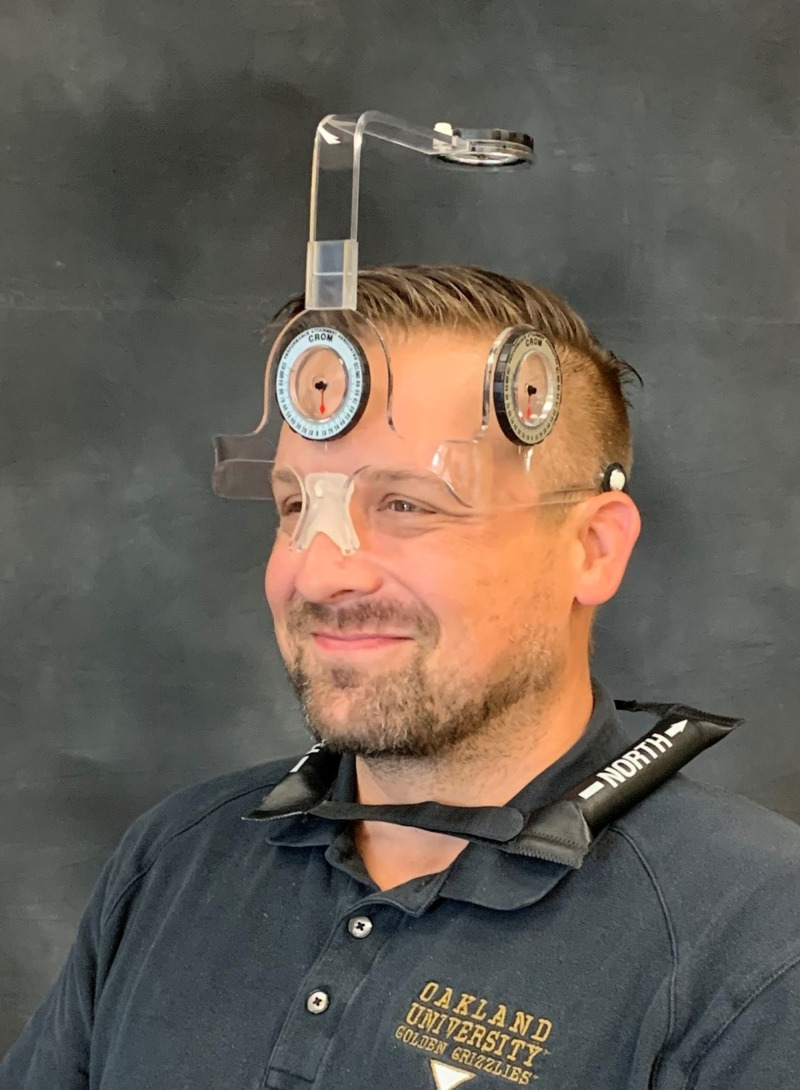
Cervical Range of Motion (CROM) Measurement Device

Cervical ROM was selected as an additional outcome measure because deficits are commonly reported for this population. These deficits are associated with impairments to functional status and, therefore, may adversely affect QoL across the survivorship continuum. Cervical ROM was measured using a cervical ROM measurement device referred to as a CROM. This device consists of a set of inclinometers placed on the head to accurately measure cervical extension and bilateral rotation (Figure 4). The CROM demonstrates good to excellent validity and reliability for rotation: right rotation, r = 0.89 (95% confidence interval, 0.81-0.94), and left rotation, r = 0.94 (95% confidence interval, 0.90-0.97). [21].

**Figure 5:**
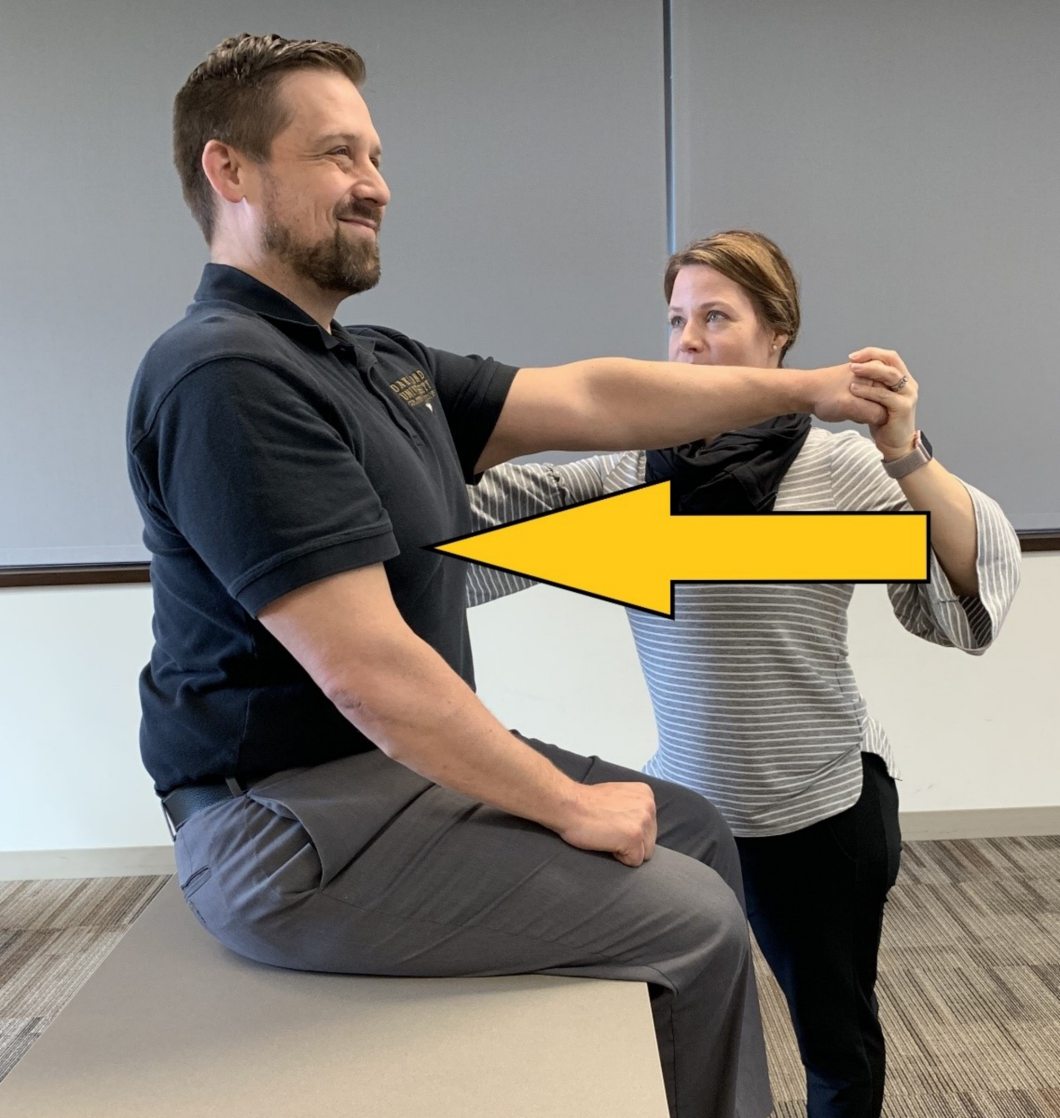
Modified Manual Muscle Testing Position for Middle Trapezius

Scapular strength measurement via manual muscle testing (MMT) was used to measure the strength of the middle trapezius muscle bilaterally due to its primary role of providing postural stability for the upper trunk, neck, and head. A modified testing position (see Figure 5), seated with shoulder flexed to 90 degrees, was selected because of anticipated post-operative positioning complications. The force was directed posteriorly through the axis of the arm. Results in this study showed that correlations between MMT and dynamometry were significant at p values less than .001. [22]. 

The sixth assessment was the 6MWT. This test is a submaximal measure of functional walking capacity at a self-selected gait speed over a six-minute time period. This test was selected due to its prevalence of use in the existing literature as well as for its ease of use in a clinical setting. The rating of perceived exertion was used to assist with gauging test fatigue. Vital statistics of blood pressure and oxygen saturation (SpO_2_) were measured throughout the test. Reliability of 6MWT was reported as r = 0.69 (p < 0.001) [23]. 

Intervention

**Figure 6:**
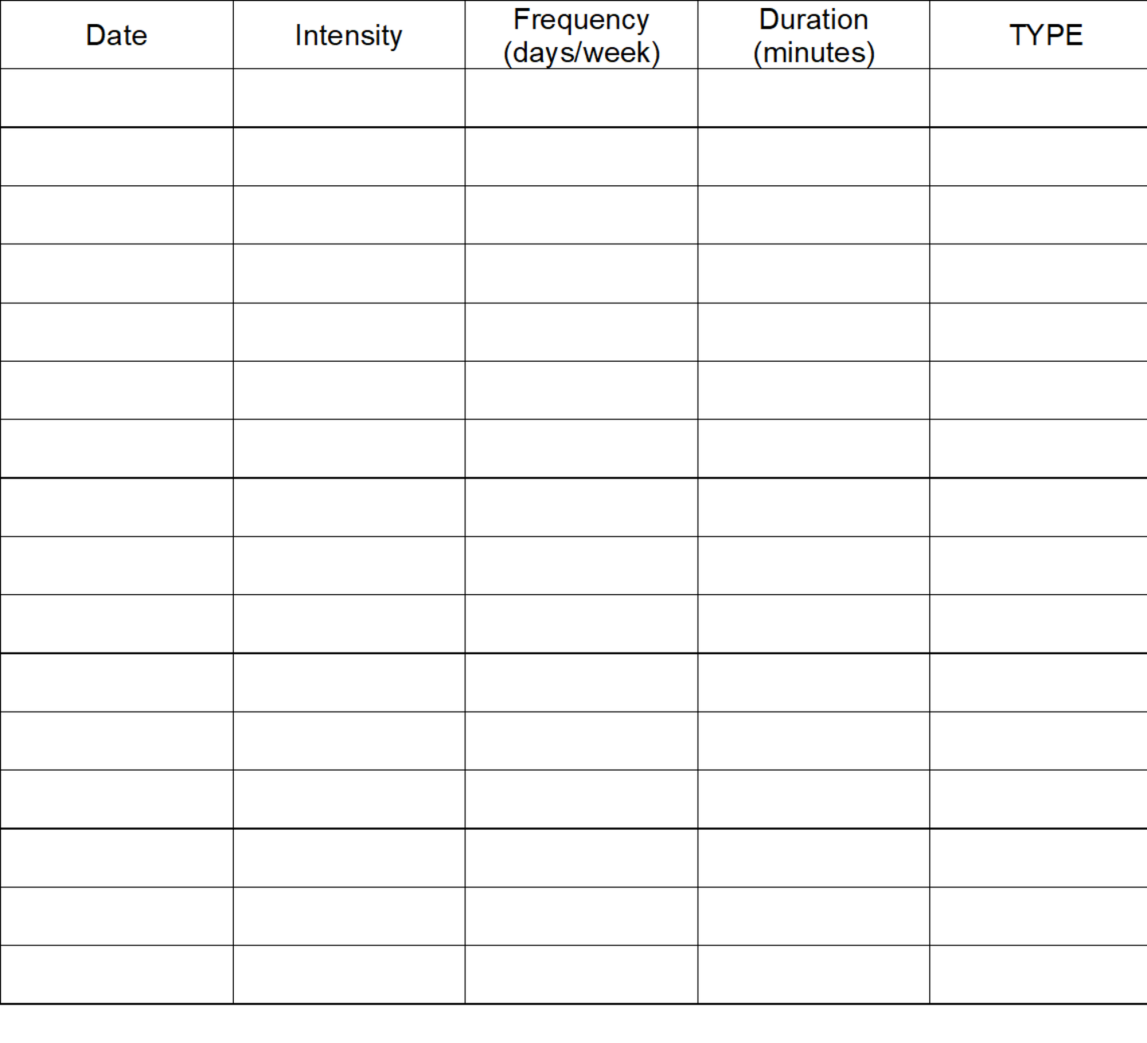
Home Exercise Program (HEP) Exercise Log

Participants received education relative to the risks and benefits of exercise and a HEP that prescribed three specific exercises. An exercise log (see Figure 6) also was provided and participants were verbally encouraged by the investigator to track frequency, duration, and type of exercise; however, use of the exercise log was not a requirement. 

**Figure 7:**
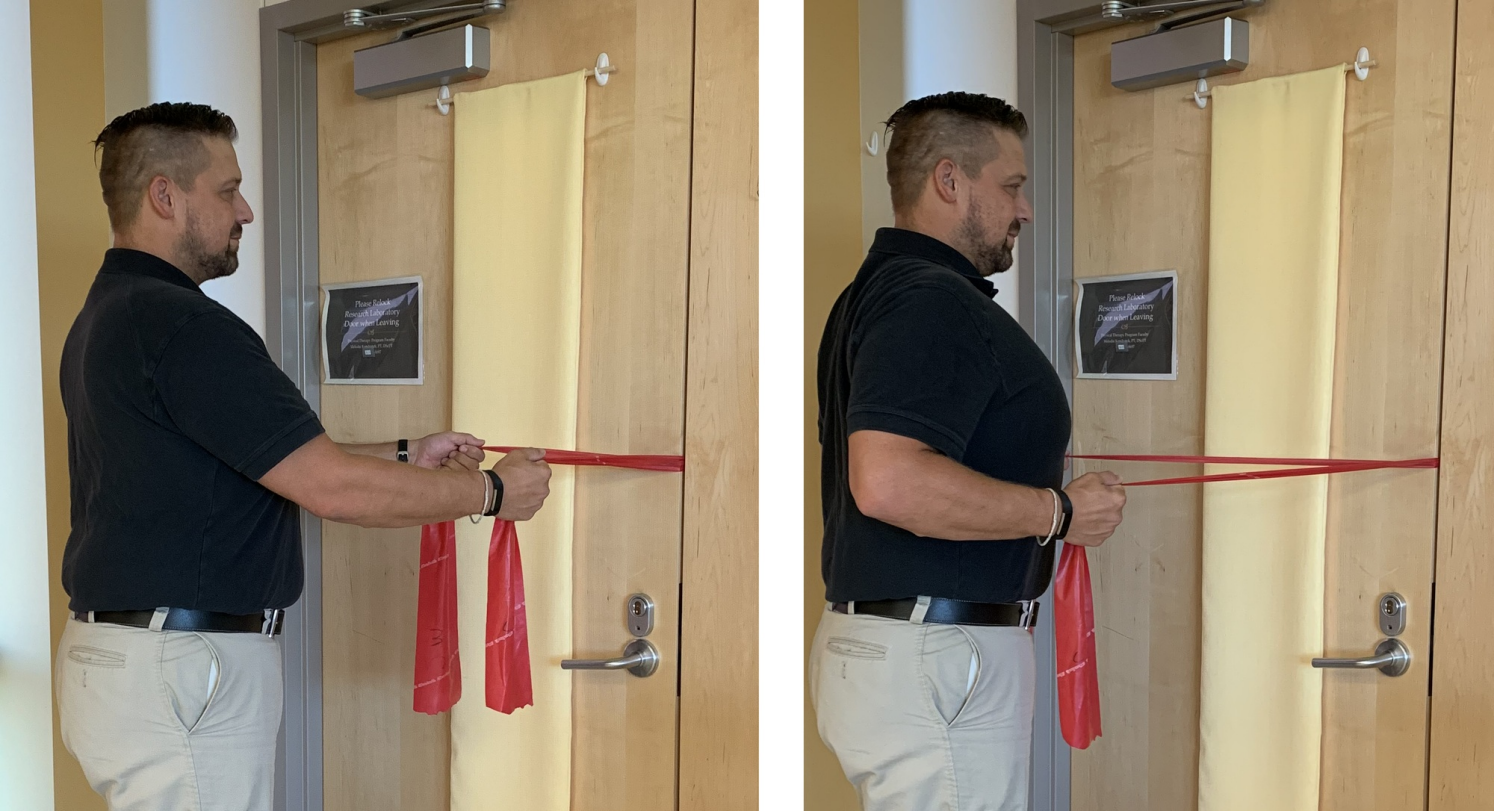
Scapular Retraction Exercise with Elastic Band Exercise begins with shoulders flexed, elbows extended and shoulder blades protracted, as shown in left pane. Exercise progresses with shoulders moving into extension, elbows flexing and shoulder blades retracting, as shown in right pane.

The first exercise in the HEP was scapular strengthening/coordination training with resistance bands (from least resistance to most: yellow, red, green, blue, black) (Figure 7). This exercise was selected because the primary action of the middle trapezius muscle is scapular retraction and it has a primary purpose in providing functional support to the upper trunk, neck, and head. All participants received red and green resistance bands with instructions to progress from red to green when they no longer were fatigued at the end of three sets of 10 repetitions. 

**Figure 8:**
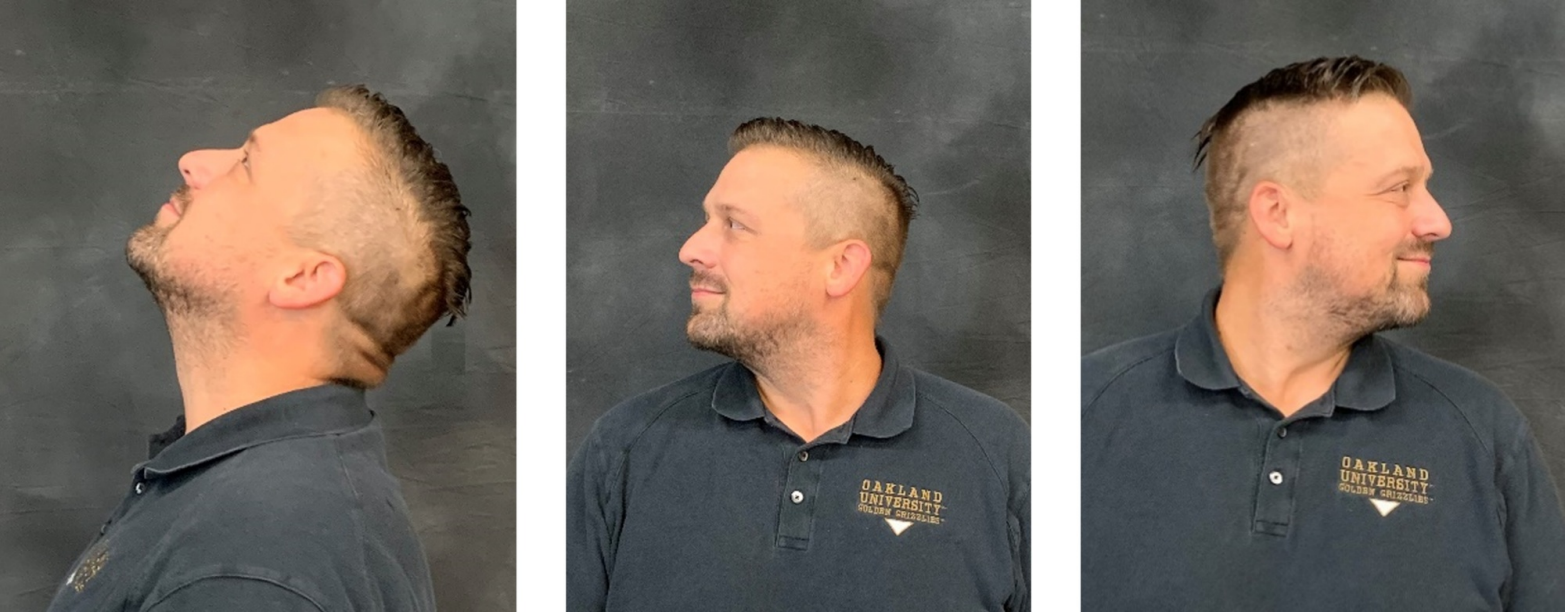
Cervical Range of Motion (CROM) Exercise Cervical extension, left pane. Cervical rotation right, middle pane. Cervical rotation left, right pane.

The second set of exercises in the HEP consisted of cervical ROM exercises including extension and bilateral rotation (Figure 8). These exercises were chosen because they facilitate maintenance of functional movement for independence with ADLs such as driving. Participants were instructed to complete one set of five repetitions for each of the cervical ROM exercises daily within their current available ROM, avoiding the onset of discomfort where applicable.

A cardiorespiratory fitness walking program was the third component of the HEP. The walking portion of the Otago exercise program was utilized for this study and consisted of instructions to warm up and cool down for two minutes each at the beginning and end of a 20-30 minute moderately-paced walk. The Otago is a structured exercise and walking program for the purpose of fall prevention [24].

Although not a formal portion of the prehabilitation protocol, patients were provided a five-day prehabilitation nutrition shake, the IMPACT-5 [25], to consume daily for the five days prior to their respective surgical procedures. The nutritional supplementation education that was an established component of this medical practice’s pre-surgical model qualifies this study as a multimodal interventional design.

Data analysis

In anticipation of a larger RCT, three participants completed all proposed study procedures. Because of the anticipated small number and limited data set for comparative analysis, descriptive statistics were utilized for this aspect of study reporting.

## Results

Three participants completed participation in the pilot testing of the protocol; their demographics are described in Table 1. All identified as Caucasian, two males and one female, with ages ranging from 60-90 years. Cancer diagnoses included squamous cell carcinoma of the tongue, mouth, and mandible. Surgical pathology ranged from stage one to four with lymph node involvement in two of the three participants. The prehabilitation period ranged from 13 to 18 days from visit one to surgery, and the entire intervention time frame from visit one to visit two ranged from 49 to 55 days. The third participant required an additional surgery secondary to not having received clear surgical margins during the first surgical intervention. 

**Table 1:** Participant Demographics and Outcomes L=left, R=right, M=Male, F=Female, SCC=Squamous Cell Carcinoma, CROM=cervical range of motion, EXT=extension, ROT=rotation, 6MWT=six minute walk test

	60 year old male	90 year old male	82 year old female
Cancer Diagnosis	SCC L lateral tongue	oral cancer	SCC R mandible
Surgical Pathology	pT2 N2B M0	pT1 pN0 Stage 1	pT4A pN2A ENE
Race	Caucasian	Caucasian	Caucasian
Days between Visit 1 to Surgery	18	13	15 (2nd surgery required 19 days after 1st surgery)
Days between Visit 1 & Visit 2	55	49	55
Change in CROM Extension	+10°	+24°	-27
Change in CROM R Rotation	0°	+6°	+1°
Change in CROM L Rotation	-6°	-38°	-22°
Change in 6MWT	-102 m	+80 m	-493 m

Health behavior questionnaire

Completion of the HBQ revealed that all participants were not engaging in regular physical activity prior to the start of the study. Reasons cited by participants included lack of time, back ache, and lack of motivation. Comprehensive results of the other HBQ questions are available in Table 2. Only one participant reported eating fruits and vegetables every day. Two of the three participants reported being non-smokers, the third participant declined to answer this question. One participant intended to address weight management in the next month, one participant was not considering change, and the third participant decline to answer. 

**Table 2:** Health Behaviors Questionnaire (HBQ) Outcomes M=male, F=female

Do you:	engage in regular physical activity?	eat 5+ servings of fruits/vegetables per day?	smoke cigarettes?	maintain a healthy weight?
M 60 Tongue Cancer	No, intend to in 6 months	Yes, > 6 months	No	No, intend in next month
M 90 Oral Cancer	No, intend to in 6 months	No, not considering change	No	No, not considering change
F 82 Mandible Cancer	No, intend to in 6 months	Not completed	Not completed	Not completed

FACT H&N

Highlights from the results of the QoL survey are listed in Table 3. Complete results are included in Tables 4-8. In the physical well-being domain, with the statement, “I have a lack of energy,” the second participant reported a lack of energy through responses of “very much” at visit one to “quite a bit” at visit two. The first and third participant’s reported energy levels remained stable. In the social/family well-being domain when responding to the statement, “I get emotional support from family,” all three participants reported receiving increased support throughout the course of the study and all three reported “very much” (the highest value) at visit two. 

**Table 3:** Functional Assessment of Cancer Therapy Head and Neck (FACT H&N) Selected Outcomes M=Male, F=Female

	M 60 Tongue Cancer		M 90 Oral Cancer		F 82 Mandible Cancer	
	Visit 1	Visit 2	Visit 1	Visit 2	Visit 1	Visit 2
Physical Well-Being Item “I have a lack of energy”	“Somewhat”	“Somewhat”	‘Very much”	“Quite a bit”	“Quite a bit”	“Quite a bit”
Social/ Family Well-Being Item “I get emotional support from my family”	“A little bit”	“Very much”	“A little bit”	“Very much”	“Quite a bit”	“Very much”
Emotional Well-Being Item “I am losing hope in the fight against my illness”	“Not at all”	“Not at all”	“Not at all”	“Not at all”	“A little bit”	“Not at all”
Functional Well-Being Item “I am content with the quality of my life right now”	“Somewhat”	“Somewhat”	“Very much”	“A little bit”	“Quite a bit”	“Not at all”
Additional Concerns Items "I can swallow naturally and easily"	“Very much”	“Somewhat”	“Very much”	“A little bit”	“Quite a bit”	“Somewhat”
“I have pain in my mouth, throat or neck”	“Not at all”	“A little bit”	“A little bit”	“A little bit”	“A little bit”	“Not at all”

**Table 4:** Functional Assessment of Cancer Therapy Head & Neck (FACT H&N) Social/Family Well-Being Outcomes

	M 60 Tongue Cancer		M 90 Oral Cancer		F 82 Mandible Cancer		
Social/ Family Well-Being Item	Visit 1	Visit 2	Visit 1	Visit 2		Visit 1	Visit 2	
“I get emotional support from my family”	“A little bit”	“Very much”	“A little bit”	“Very much”		“Quite a bit”	“Very much”	
“I get support from my friends”	“A little bit”	“Very much”	“Very much”	“Very much”		“Very much”	“Very much”	
“My family has accepted my illness”	“Somewhat”	“Quite a bit”	“Very much”	“Very much”		“Very much”	“Very much”	
“I am satisfied with family communication about my illness”	“Very much”	“Very much”	“Very much”	“Very much”		“Quite a bit”	“Very much”	
“I feel close to my partner” (or person who is main support)	“Very much”	“Very much”	“Not at all”	“Very much”		“Not at all”	“Very much”	

**Table 5:** Functional Assessment of Cancer Therapy Head & Neck (FACT H&N) Emotional Well-Being Outcomes M=male, F=female

	M 60 Tongue Cancer		M 90 Oral Cancer			F 82 Mandible Cancer	
Emotional Well-Being Item	Visit 1	Visit 2	Visit 1	Visit 2	Visit 1		Visit 2
“I am satisfied with how I am coping with my illness”	“Quite a bit”	“Very much”	“Very much”	“Quite a bit”		“Somewhat”	“Somewhat”
“I am losing hope in the fight against my illness”	“Not at all”	“Not at all”	“Not at all”	“Not at all”		“A little bit”	“Not at all”
“I worry about dying”	“A little bit”	“Not at all”	“Not at all”	“Not at all”		“A little bit”	“Not at all”
“I worry that my condition will get worse”	“Not at all”	“A little bit”	“Not at all”	“Not at all”		“A little bit”	“Somewhat”

**Table 6:** Functional Assessment of Cancer Therapy Head & Neck (FACT H&N) Physical Well-being Outcomes M=male, F=female

	M 60 Tongue Cancer		M 90 Oral Cancer		F 82 Mandible Cancer	
Physical Well-Being Item	Visit 1	Visit 2	Visit 1	Visit 2	Visit 1	Visit 2
“I have a lack of energy”	“Somewhat”	“Somewhat”	‘Very much”	“Quite a bit”	“Quite a bit”	“Quite a bit”
“I am bothered by the side effects of treatment”	“Not at all”	“Somewhat”	“Not at all”	“Not at all”	”Not at all”	“Somewhat”

**Table 7:** Functional Assessment of Cancer Therapy Head & Neck (FACT H&N) Functional Well-Being Outcomes M=Male, F=Female

	M 60 Tongue Cancer		M 90 Oral Cancer		F 82 Mandible Cancer	
Functional Well-Being Item	Visit 1	Visit 2	Visit 1	Visit 2	Visit 1	Visit 2	
“I am able to work” (Includes work at home)	“Quite a bit”	“Somewhat”	“Quite a bit”	“A little bit”	“Quite a bit”	“Not at all”	
“I have accepted my illness”	“Very much”	“Very much”	“Very much”	“Quite a bit”	“Quite a bit”	“Quite a bit”	
“I am sleeping well”	“Not at all”	“A little bit”	“Not at all”	“Very much”	“Somewhat”	“Quite a bit”	
“I am content with the quality of my life right now”	“Somewhat”	“Somewhat”	“Very much”	“A little bit”	“Quite a bit”	“Not at all”	

**Table 8:** Functional Assessment of Cancer Therapy Head & Neck (FACT H&N) Items of Additional Concern Outcomes M=Male, F=Female

	M 60 Tongue Cancer		M 90 Oral Cancer		F 82 Mandible Cancer	
Additional Concerns Item	Visit 1	Visit 2	Visit 1	Visit 2	Visit 1	Visit 2
“I am able to eat the foods that I like”	“Very Much”	“Very Much”	“Very Much”	“A little bit”	“Quite a bit”	“Not at all”
“My mouth is dry”	“Very much”	“Quite a bit”	“Somewhat”	“Quite a bit”	“Quite a bit”	“Quite a bit”
“I have trouble breathing”	“A little bit”	“Not at all”	“Not at all”	“Not at all”	“A little bit”	“A little bit”
“My voice has its usual quality and strength”	“Very much”	“Very much”	“Very much”	“Somewhat”	“Quite a bit”	“A little bit”
“I am unhappy how my face and neck look”	“Not at all”	“A little bit”	“Not at all”	“Not at all”	“Somewhat”	“A little bit”
"I can swallow naturally and easily"	“Very much”	“Somewhat”	“Very much”	“A little bit”	“Quite a bit”	“Somewhat”
“I am able to communicate with others”	“Very much”	“Very much”	“Very much”	“Very much”	“Quite a bit”	“Somewhat”
“I have pain in my mouth, throat or neck”	“Not at all”	“A little bit”	“A little bit”	“A little bit”	“A little bit”	“Not at all”

In the domain of emotional well-being and in response to the statement of “I am losing hope in the fight against my illness,” all three participants reported increased hope throughout the duration of the study. The first and second participants expressed no loss of hope and the third participant became more hopeful, moving from “a little bit” at visit one to “not at all” at visit two. In the functional well-being domain, all three participants reported diminished ability to work throughout the course of the study, which encompassed work to be done around the home. In response to the statement, “I am content with the quality of my life right now,” the second and third participants reported a decline in QoL, with participant two moving from “very much” to “a little bit” from visit one to visit two and participant three moving from “Quite a bit” to “Not at all.” The first participant reported unchanged perception of QoL with a response of “somewhat” at visit one and two.

Relative to items of additional concern on the QoL survey and in response to the statement, “I can swallow naturally and easily,” all three participants reported diminished ease of swallowing function from visit one to visit two. Relative to their pain experience, all three participants reported a little bit of pain or less throughout the course of treatment. The first participant, however, reported increased pain. The second participant reported pain that was unchanged, and the third participant reported improved pain throughout the course of the study. 

Cervical range of motion and strength

Summarized results of the CROM assessment are listed in Table 1. Complete results are included in Table 9. The first and third participants demonstrated a loss of cervical extension and bilateral rotation from visit one to visit two, 10 and 6 degrees respectively. Interestingly, one male participant demonstrated an improvement in cervical rotation by 6 degrees to the left and 1 degree to the right. All three participants maintained middle trapezius strength throughout the duration of the study (Table 10).

**Table 9:** Cervical Range of Motion (CROM) Outcomes M=Male, F=Female

	Extension			Left Rotation			Right Rotation		
Participant	Visit 1	Visit 2	Degrees of change	Visit 1	Visit 2	Degrees of change	Visit 1	Visit 2	Degrees of change
M 60 Tongue Cancer	51°	41°	-10°	77°	53°	-24°	80°	53°	-27°
M 90 Oral Cancer	55°	55°	0°	52°	58°	+6°	58°	59°	+1°
F 82 Mandible Cancer	43°	37°	-6°	80°	42°	-38°	64°	42°	-22°

**Table 10:** Scapular Strength via Manual Muscle Testing (MMT) Outcomes

Participant	Visit 1	Visit 2	Change
60 y.o. Male Tongue Cancer	5	5	0
90 y.o. Male Oral Cancer	5	5	0
82 y.o. Female Mandible Cancer	5	5	0

6 Minute Walk Test

Summarized results of the 6MWT are listed in Table 1. Complete results are included in Table 11. The first and third participants experienced a decline in walking distance over the course of the study by 102 feet and 493 feet respectively, whereas, the second participant experienced an increase of 80 feet. Age- and sex-adjusted normative values for the 6MWT are indicated within Table 11 [26]. Two participants were observed to have initiated the study with below average values and the other had values within the normal range for age and sex. 

**Table 11:** Six Minute Walk Test (6MWT) Outcomes M=Male, F=Female

Participant	M 60 Tongue Cancer			M 90 Oral Cancer			F 82 Mandible Cancer		
	Visit 1	Visit 2	Change	Visit 1	Visit 2	Change	Visit 1	Visit 2	Change
Walk Test Results (in feet)	1372	1270	-102	520	600	80	918	425	-493
Age & Sex Related Norms [26]		1634			971			922

## Discussion

The primary purpose of this study was to describe the development of a multimodal prehabilitation study protocol. The secondary purpose was to report feasibility study results of the study protocol from patients diagnosed with H&N cancer. The tertiary purpose was to describe the evolution of the protocol for the pilot phase.

This feasibility study adds to the body of evidence for PT-guided prehabilitation interventions among cancer survivors. The investigators believe this to be the first H&N prehabilitation protocol designed to encompass commonly used PT assessments and interventions. In addition, this is the first study completed using this new intervention design.The results of this study are beneficial for trend identification and for informing future research in this domain. Although a completed pilot RCT using an adequately powered study design is warranted, initial participant outcomes suggest that this sample was sedentary, nutritionally compromised, and overweight. All participants maintained pre-surgical strength throughout the post-surgical trajectory despite advanced age. Two of the three participants experienced reduced cervical ROM into extension and bilateral rotation despite the provided interventions, speaking to the significant consequences of immobility of the surgical area in the immediate post-operative period. As there was not a comparable control group to examine differences in post-operative CROM outcomes, patients who did not receive the prehabilitation interventions may have demonstrated a larger loss of cervical ROM. 

Treatment for H&N cancer also negatively impacted functional walking capacity in spite of the intervention for two of the three participants, though comparison to normative values is revealing in that both male participants began the study significantly below values for age-matched peers. The female participant began the study within the range of normal when age matched to her peers, but her walking capacity may have been further impacted because she required two surgical procedures and subsequent hospitalizations within the study time frame. In comparison to the existing literature, this pilot study data deviates somewhat in that most of the studies reviewed to inform this project noted improved distances for the 6MWT and, therefore, for functional walking capacity. This deviation can perhaps be accounted for by the advanced age of two of the three participants and the increased impact of physiological decline as a result of the stressors of H&N cancer surgery for these participants.

Relative to QoL trends, all three participants reported improved emotional support from family members throughout the course of treatment. Similarly, two of the three participants reported feeling increased closeness with their partner, with all three participants reporting feeling “very much” closeness (the highest value) with their partner. This finding speaks to the increased bonding and closeness that often develops in a family unit throughout a cancer journey and is supported by existing literature [27]. Physical therapists may facilitate this bonding by communicating openly, respecting the wishes of the patient, and engaging all appropriate family members in the care delivery process as warranted [28]. In addition, PTs may be able to engage family members in assisting with healthy behavior change in light of this new closeness. Furthermore, all three participants reported diminished ability to work, including work around the home, highlighting the significant global impact of cancer treatment adverse effects and the need for effective intervention before, during, and after cancer treatment. 

Suggestions for future research

Suggestions for future research include the use of multiple surgeons/institutions to improve recruitment and generalizability. Additionally, a second pre-operative visit may be beneficial to reinforce exercise compliance and to evaluate clinical outcomes before substantive changes after surgery. A simplified HEP is suggested to improve participant understanding and, therefore, compliance. Use of handheld dynamometry is suggested to replace MMT as the measure of strength. Grip strength has been found in the literature to be a useful proxy measure of general strength [29]. Additionally, the use of the Two Minute Walk Test (2MWT) is suggested to replace the 6MWT to assess cardiorespiratory fitness to minimize the burden in busy clinic settings and is cited in the literature as a suitable alternative [30].

## Conclusions

This newly developed prehabilitation protocol is feasible to implement and can be replicated at other health care institutions. Prehabilitation for patients diagnosed with H&N cancer has the potential to mitigate treatment adverse effects and to improve QoL across the cancer survivorship continuum. The use of CROM, scapular strength, and timed walking tests as outcome measures yielded promising results during pilot testing. Further investigation of the efficacy of prehabilitative education and exercise for persons diagnosed with H&N cancers is warranted.
